# Draft Genome Sequence of an Extracellular Protease-Producing Bacterium, *Stenotrophomonas bentonitica* VV6, Isolated from Arctic Seawater

**DOI:** 10.1128/genomeA.01610-17

**Published:** 2018-02-22

**Authors:** Ming-Xia Chen, He-Yang Li, Xiao-Sheng Ye, Xiao-Yu He

**Affiliations:** a Department of Bioengineering and Biotechnology, Huaqiao University, Fujian, Xiamen, China; b Third Institute of Oceanography, State Oceanic Administration, Fujian, Xiamen, China

## Abstract

The draft genome sequence of the extracellular protease-producing bacterium *Stenotrophomonas bentonitica* VV6, isolated from Arctic seawater, was established. The genome size was approximately 4.365 Mb, with a G+C content of 66.54%, and it contains 3,871 predicted protein-coding sequences (CDSs) and 60 tRNAs.

## GENOME ANNOUNCEMENT

To date, the genus *Stenotrophomonas* comprises 15 species with validly published names isolated from diverse environments, being mostly associated with plants and soil (1). Strain VV6 is an extracellular protease-producing *Stenotrophomonas* bacterium isolated from Arctic seawater (53.328499°N, 169.960434°E, 1,937 m depth) (8°C, with salinity 32.9, as of 10 July 2010). Optimal growth occurs at 27.5°C to 32.5°C, at pH 6.0, and with 2% to 3% (wt/vol) NaCl. In order to obtain the cold-active industrial enzymes and study the evolutionary mechanism of cold adaptation of mesophilic bacteria, strain VV6 was chosen for whole-genome sequencing.

The draft genome sequence of strain VV6 was performed by Shanghai Majorbio Bio-pharm Technology (Shanghai, China), using Solexa paired-end (400-bp library) sequencing technology. For VV6, 1,368 Mbp of clean data were generated to reach about 346-fold depth of coverage, as determined using an Illumina/Solexa Genome Analyzer IIx (Illumina). The clean data were assembled by SOAP*denovo*2 (2). The final assemblies of strain VV6 resulted in 45 contigs, with a total size of 4,364,949 bp, G+C content of 66.54%, *N*_50_ value of 400,665 bp, and *N*_90_ value of 90,712 bp.

The 16S rRNA gene (1,506 bp) sequence is 99.86% identical to that of *Stenotrophomonas bentonitica* BII-R7^T^, followed by that of *Stenotrophomonas rhizophila* DSM 14405^T^ (99.39%) and that of *Stenotrophomonas maltophilia* MTCC 434^T^ (98.5%). The average nucleotide identity (ANI) was calculated using the algorithm described by Yoon et al. (3) with the web service EzBioCloud. The draft genome of strain VV6 is 97.54% identical to that of *S. bentonitica* PML168 (the genome of type strain BII-R7 was not available at the time), 86.13% identical to that of *S. rhizophila* DSM 14405^T^, and 81.56% identical to that of *S. maltophilia* ATCC 13637^T^. The digital DNA-DNA hybridization (dDDH) was determined using the Genome-to-Genome Distance Calculator (GGDC) version 2.0 (http://ggdc.dsmz.de/distcalc2.php), as described by Meier-Kolthoff et al. (4). The dDDH estimated values between strain VV6 and *S. bentonitica* PML168, *S. rhizophila* DSM 14405^T^, and *S. maltophilia* ATCC 13637^T^ are 78.8%, 31%, and 24.6%, respectively. Having both ANI values bigger than 95% (5) and dDDH value bigger than 70% (6) indicates that strain VV6 and *S. bentonitica* PML168 belong to the same species. *S. bentonitica* is a recently described high-uranium-tolerant and high-selenium-tolerant strain that was isolated from bentonite formations in Spain (7, 8).

Gene annotation was carried out using the NCBI Prokaryotic Genome Annotation Pipeline (PGAP) (9). In total, 3,871 protein-coding sequences (CDSs) and 60 tRNAs were predicted. Among the CDSs, 2,908 and 1,999 proteins could be assigned to COG/KOG/NOGs families and KEGG orthologous groups, respectively. The genomic analysis identified genes responsible for industrially important enzymes, such as protease, esterase/lipase, chitinase, amylase, cellulase, catalase, oxidase, glucanase, polysaccharide deacetylase, glycosyltransferase, alcohol dehydrogenase, and alkaline phosphatase. β-Lactamases were produced by VV6 which provide multiresistance to β-lactam antibiotics, such as penicillins, cephalosporins, and cephamycins. Cold shock proteins, which regulate the expression of various genes, including those involved in stress resistance and virulence of bacteria (10), were also found. Comparative genomic sequence analysis between strain VV6 and other strains of *S. bentonitica* from different environments will provide further insight into the mechanism of cold adaptation and allow a more comprehensive characterization of this bacterium.

### Accession number(s).

This whole-genome shotgun project has been deposited in DDBJ/ENA/GenBank under the accession no. PKQP00000000. The version described in this paper is version PKQP01000000.
